# Angiotensin II receptor blockade promotes repair of skeletal muscle through down-regulation of aging-promoting C1q expression

**DOI:** 10.1038/srep14453

**Published:** 2015-09-25

**Authors:** Chizuru Yabumoto, Hiroshi Akazawa, Rie Yamamoto, Masamichi Yano, Yoko Kudo-Sakamoto, Tomokazu Sumida, Takehiro Kamo, Hiroki Yagi, Yu Shimizu, Akiko Saga-Kamo, Atsuhiko T. Naito, Toru Oka, Jong-Kook Lee, Jun-ichi Suzuki, Yasushi Sakata, Etsuko Uejima, Issei Komuro

**Affiliations:** 1 Department of Cardiovascular Medicine, Osaka University Graduate School of Medicine, Suita, Osaka 565-0871, Japan; 2 Clinical Pharmacy Education Unit, Graduate School of Pharmaceutical Sciences, Osaka University, Suita, Osaka 565-0871, Japan; 3 Department of Cardiovascular Medicine, Graduate School of Medicine, The University of Tokyo, Bunkyo-ku, Tokyo 113-8655, Japan; 4 Department of Cardiovascular Medicine, Chiba University Graduate School of Medicine, Chiba, Chiba 260-8670, Japan; 5 Department of Cardiovascular Regenerative Medicine, Osaka University Graduate School of Medicine, Suita, Osaka 565-0871, Japan; 6 Department of Advanced Clinical Science and Therapeutics, Graduate School of Medicine, The University of Tokyo, Bunkkyo-ku, Tokyo 113-8655, Japan; 7 AMED-CREST, Japan Agency for Medical Research and Development, Chiyoda-ku, Tokyo 100-0004, Japan

## Abstract

Disruption of angiotensin II type 1 (AT_1_) receptor prolonged life span in mice. Since aging-related decline in skeletal muscle function was retarded in *Atgr1a*^−/−^ mice, we examined the role of AT_1_ receptor in muscle regeneration after injury. Administration of AT_1_ receptor blocker irbesartan increased the size of regenerating myofibers, decreased fibrosis, and enhanced functional muscle recovery after cryoinjury. We recently reported that complement C1q, secreted by macrophages, activated Wnt/β-catenin signaling and promoted aging-related decline in regenerative capacity of skeletal muscle. Notably, irbesartan induced M2 polarization of macrophages, but reduced C1q expression in cryoinjured muscles and in cultured macrophage cells. Irbesartan inhibited up-regulation of *Axin*2, a downstream gene of Wnt/β-catenin pathway, in cryoinjured muscles. In addition, topical administration of C1q reversed beneficial effects of irbesartan on skeletal muscle regeneration after injury. These results suggest that AT_1_ receptor blockade improves muscle repair and regeneration through down-regulation of the aging-promoting C1q-Wnt/β-catenin signaling pathway.

Angiotensin (Ang) II is the crucial bioactive molecule of the renin-angiotensin system, and exerts most of its pathophysiological actions through binding to Ang II type 1 (AT_1_) receptor^1^,^2^. While humans have a single *AGTR1* gene that encodes AT_1_ receptor, mice have *Agtr1a* and *Agtr1b* genes encoding two isoforms (AT_1a_ and AT_1b_) of AT_1_ receptor, and the major isoform of mouse AT_1_ receptor is AT_1a_ receptor^1^,^2^. AT_1_ receptor is a member of the G protein-coupled receptor family, which shares typical conformation of seven transmembrane-spanning α-helices^3^. Upon stimulation by binding to Ang II or mechanical stretch^4^,^5^, AT_1_ receptor activates multiple G protein-dependent and –independent signaling pathways and promote the formation of reactive oxygen species (ROS), leading to a variety of responses such as cellular hypertrophy and proliferation, vascular contraction, inflammatory responses, and salt and water retention^6^,^7^.

Besides homeostatic regulation of blood pressure and electrolyte and water balance, AT_1_ receptor plays physiological roles in normal organ development. Targeted disruption of both *Agtr1a* and *Agtr1b* genes in mice resulted in abnormal kidney development characterized by tubular atrophy and interstitial expansion, papillary atrophy, and severe impairment of concentrating urine^8^,^9^. In this sense, AT_1_ receptor is essential during embryogenesis and beneficial for organismal survival. However, sustained and excessive activation of AT_1_ receptor is detrimental, promoting various aging-related diseases such as cardiovascular diseases, diabetes, chronic kidney disease, dementia, osteoporosis, and cancer^10^,^11^,^12^,^13^,^14^. Furthermore, recent studies unraveled the involvement of AT_1_ receptor in aging process *per se*^15^,^16^. Targeted disruption of *Agtr1a* gene prolonged life span in mice, which was associated with prevention of aging-related progression of cardiac hypertrophy and fibrosis^17^. Accordingly, AT_1_ receptor plays antagonistic and pleiotropic roles according to the ages and pathophysiological conditions^14^.

Benigni A. *et al.* reported that *Agtr1a*^−/−^ mice showed a decrease in oxidative stress, an increase in mitochondrial number, and an increase in expression levels of prosurvival genes such as *Nampt* and *Sirt3*, which in combination might contribute to a prolongation of life span^17^. However, it remains precisely unknown how AT_1_ receptor regulates life span. We observed that aging-related decline in skeletal muscle function was prominently milder in *Agtr1a*^−/−^ mice, as compared with *Agtr1a*^+/+^ littermate mice, and that systemic administration of an AT_1_ receptor blocker (ARB) irbesartan significantly enhanced muscle weight recovery after cryoinjury in wild-type mice. Mechanistically, AT_1_ receptor blockade down-regulated the aging-promoting C1q-Wnt/β-catenin signaling pathway. We recently reported that complement C1q activated β-catenin signaling independently of Wnts, and promoted impairment of skeletal muscle regeneration with aging^18^. Our study provides evidence for the hierarchical relationship between AT_1_ receptor signaling and C1q/Wnt-β-catenin signaling in promoting aging-related functional decline, and indicates that AT_1_ receptor blockade emerges as a preventive strategy against geriatric sarcopenia and frailty.

## Results

### Aging-related decline in skeletal muscle function was milder in *Agtr1a* ^−/−^ mice.

First, we performed a prospective observational study in 66 *Agtr1a*^*−/−*^ mice and 55 *Agtr1a*^*+/+*^ littermate mice, and found that *Agtr1a*^*−/−*^ mice significantly lived longer than *Agtr1a*^*+/+*^ mice (Supplementary Fig. 1) as previously reported^17^. The average life span of *Agtr1a*^−/−^ and *Agtr1a*^+/+^ mice was 760.0 ± 20.9 and 651.8 ± 21.7 days, respectively (*P* < 0.05). In association with the elongation of life span, aging-related changes were significantly milder in multiple tissues of *Agtr1a*^−/−^ mice. For example, aging-related thinning of epidermal and fat layers was milder in *Agtr1a*^−/−^ mice (Supplementary Fig. 2a). After shaving of dorsal hair in the same area, hair growth was significantly more rapid in *Agtr1a*^−/−^ mice than in *Agtr1a*^+/+^ mice at the age of 18 months, although it was comparable at the age of 3 months (Supplementary Fig. 2b). Especially, aging-related changes in locomotive activity and coordination skill were milder in *Agtr1a*^−/−^ mice. In both vertical pole test and hanging wire test, the time to fall off was significantly longer in *Agtr1a*^−/−^ mice at the age of 12 and 18 months, although it was comparable at the age of 3 months (Supplementary Fig. 3a,b). In addition, aging-associated fibrosis in skeletal muscle at the age of 24 months was significantly less severe in *Agtr1a*^−/−^ mice, as revealed by Masson’s trichrome staining (Supplementary Fig. 3c). These results suggest that aging-related decline in skeletal muscle function is milder in *Agtr1a*^−/−^ mice.

### Irbesartan enhanced repair and functional recovery of skeletal muscle after cryoinjury

Since impairment of regeneration potential is one of the most important features observed during aging of skeletal muscles^19^, we investigated the role of AT_1_ receptor signaling in skeletal muscle regeneration in response to injury. We previously reported that AT_1_ receptor signaling regulates the hypothalamic neurocircuit that is involved in the control of food intake, and that *Agtr1a*^−/−^ mice were hyperphagic and obese with increased adiposity on an *ad libitum* diet, as compared with *Agtr1a*^+/+^ mice^20^. To avoid the metabolic effects on skeletal muscle regeneration in *Agtr1a*^−/−^ mice, we cryoinjured tibialis anterior (TA) muscle of wild-type mice and examined the effects of treatment with an AT_1_ receptor blocker irbesartan on muscle regeneration after injury. We first confirmed that orally administered irbesartan (20 mg/kg/d) had no effect on daily food intake and body weight (Supplementary Fig. 4), as well as heart rate and blood pressure (Supplementary Table 1).

**Figure 1:**
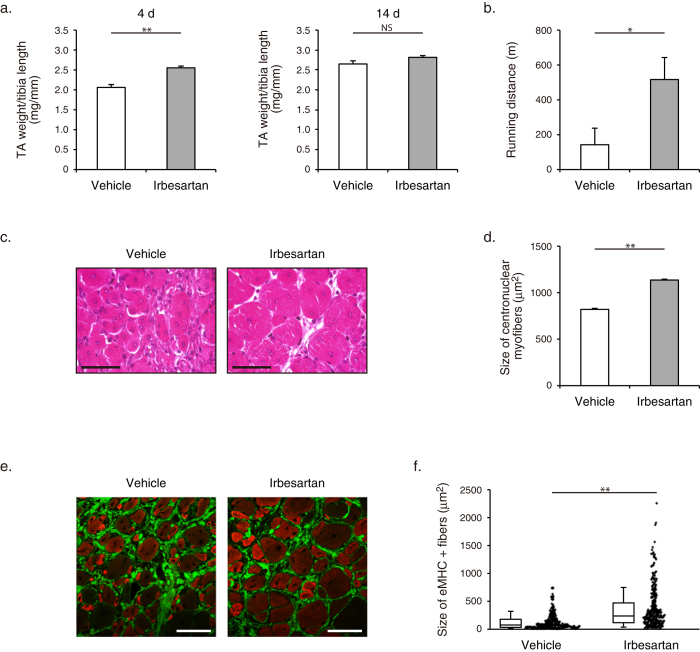
Functional and histological recovery of skeletal muscle after cryoinjury in irbesartan-treated mice. (**a**) TA weight-to-tibia length ratios in mice treated with irbesartan or vehicle at 4 d (*n* = 5, in each group) and 14 d (*n* = 4, in each group) after cryoinjury. Data are presented as mean ± SEM. **P < 0.01. NS, not significant. (**b**) Total running distance during treadmill test in mice treated with irbesartan or vehicle at 14 d after cryoinjury (*n* = 7, in each group). Data are presented as mean ± SEM. *P < 0.05. (**c**) Histological sections with hematoxylin and eosin (HE) staining of TA muscles in mice treated with irbesartan or vehicle at 14 d after cryoinjury. Scale bars, 50 μm. (**d**) The sizes of centronuclear myofibers in TA muscles of mice treated with irbesartan or vehicle (irbesartan, *n* = 2,062; vehicle, *n* = 1,943) at 14 d after cryoinjury. Data are presented as mean ± SEM. **P < 0.01. (**e**) Immunostaining of TA muscles of mice treated with irbesartan or vehicle at 10 d after cryoinjury. Embryonic myosin heavy chain (eMHC) and Collagen 1 are represented in red and green, respectively. Scale bar, 50 μm. (**f**) Scatter plot and box plot of the sizes of eMHC + fibers in TA muscles of mice treated with irbesartan or vehicle at 10 d after cryoinjury (*n* = 6 sections from 3 mice in each group). **P < 0.01.

**Figure 2:**
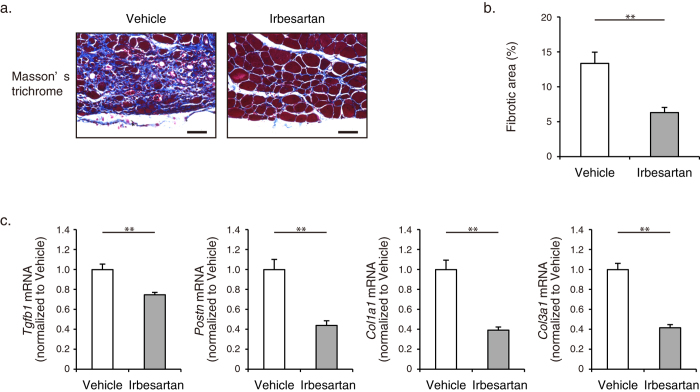
Fibrosis in cryoinjured skeletal muscle of irbesartan-treated mice. (**a**) Histological sections with Masson’s trichrome staining of TA muscles in mice treated with irbesartan or vehicle at 14 d after cryoinjury. Scale bars, 50 μm. (**b**) The percent area of fibrosis in Masson’s trichrome staining of TA muscles in mice treated with irbesartan or vehicle at 14 d after cryoinjury (*n* = 5, in each group). Data are presented as mean ± SEM. **P < 0.01. (**c**) The mRNA levels of *Tgfb1*, *Postn*, *Col1a1*, and *Col3a1* in TA muscles of mice treated with irbesartan or vehicle at 10 d after cryoinjury (*n* = 8, in each group). Data are shown as fold induction over vehicle (mean ± SEM). **P < 0.01.

We next compared repair and functional recovery of skeletal muscle between irbesartan- and vehicle-treated mice. Irbesartan-treated mice showed a significantly higher weight of TA muslce than vehicle-treated mice at 4 d after cryoinjury, although the TA weight-to-tibia length ratios were comparable at 14 d (Fig. 1a). Total running distance during treadmill testing at 14 d was significantly longer in irbesartan-treated mice than in vehicle-treated mice (Fig. 1b). Histological analysis revealed that the size of centronuclear regenerating myofibers at 14 d was significantly larger in irbesartan-treated mice than in vehicle-treated mice (Fig. 1c,d). In addition, the number and size of embryonic myosin heavy chain (eMHC)-positive myofibers at 10 d were significantly increased in irbesartan-treated mice, as compared with vehicle-treated mice (Fig. 1e,f). Fibrotic area in skeletal muscle at 14 d after injury was significantly smaller in irbesartan-treated mice than in vehicle-treated mice, as revealed by Masson’s trichrome staining (Fig. 2a,b). qRT-PCR analysis also showed a significant decrease in mRNA expression of fibrosis-related genes such as *Tgfb1*, *Postn*, *Col1a1*, and *Col3a1* in TA muscles of irbesartan-treated mice at 10 d after injury, as compared with vehicle-treated mice (Fig. 2c). These results suggest that AT_1_ receptor blockade promotes myogenic growth after injury.

### Irbesartan increased Pax7 + satellite cells in regenerating skeletal muscle after cryoinjury

**Figure 3:**
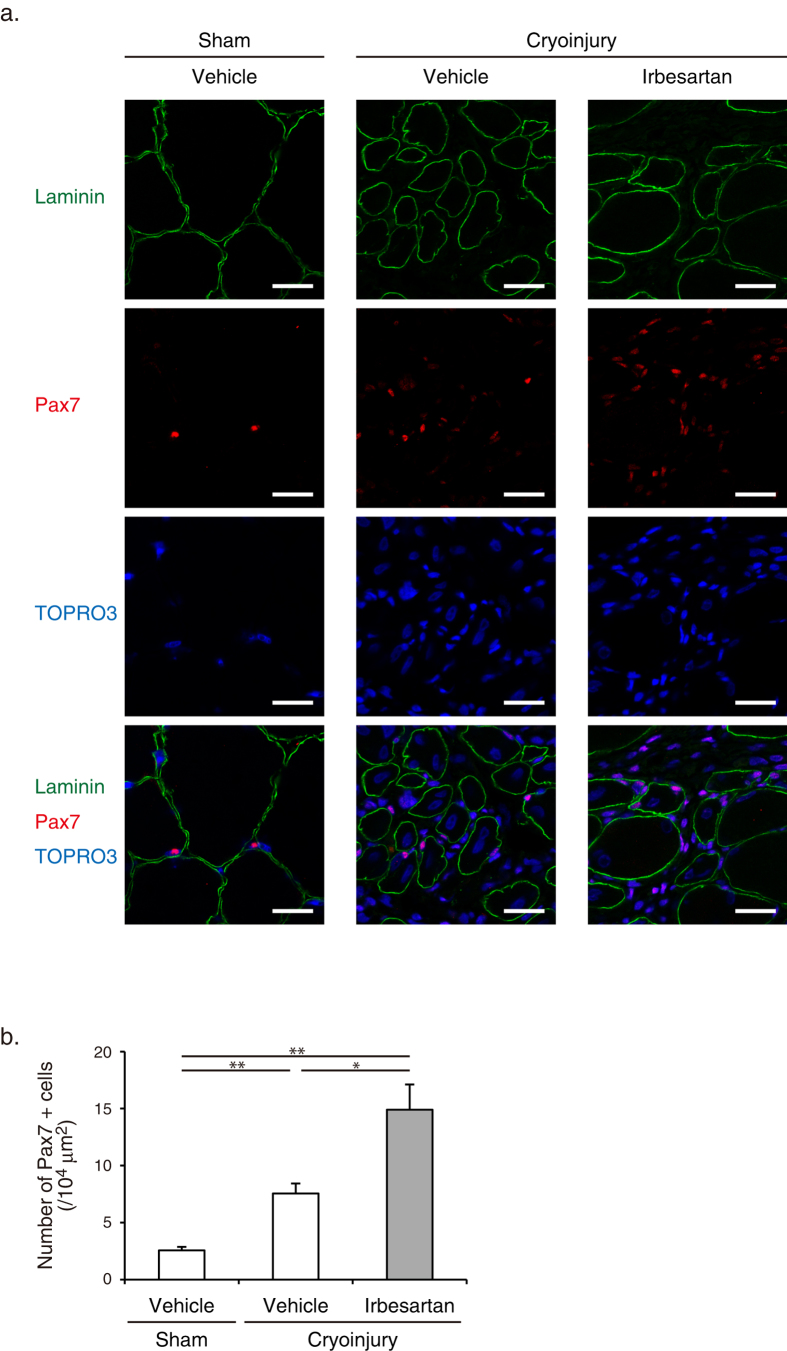
Pax7 + satellite cells in cryoinjured skeletal muscle of irbesartan-treated mice. (**a**) Immunostaining of TA muscles of mice treated with irbesartan or vehicle at 4 d after cryoinjury or sham operation. Laminin, Pax7 are represented in green and red, respectively, with TO-PRO-3 staining of the nucleus (blue). Scale bar, 20 μm. (**b**) The number of Pax7 + satellite cells in TA muscles of mice treated with irbesartan or vehicle at 4 d after cryoinjury or sham operation (*n* = 11, in each group). Data are presented as mean ± SEM. *P < 0.05, **P < 0.01.

Satellite cells, characterized by the expression of the paired box protein Pax7, are crucial for skeletal muscle growth and regeneration^19^. In resting muscles, satellite cells are located juxtaposed to muscle fibers in a quiescent state, but re-enter the cell cycle and proliferate to form sufficient number of myofibers in response to injury^19^. Immunohistochemical analysis revealed that the number of Pax7 + satellite cells was significantly increased in TA muscles at 4 d after cryoinjury of irbesartan-treated mice, as compared with vehicle-treated mice (Fig. 3). These results suggest that AT_1_ receptor blockade promotes activation and cell cycle re-entry of Pax7 + satellite cells following injury.

### Irbesartan induced M2 polarization of macrophages in regenerating skeletal muscle after cryoinjury

**Figure 4:**
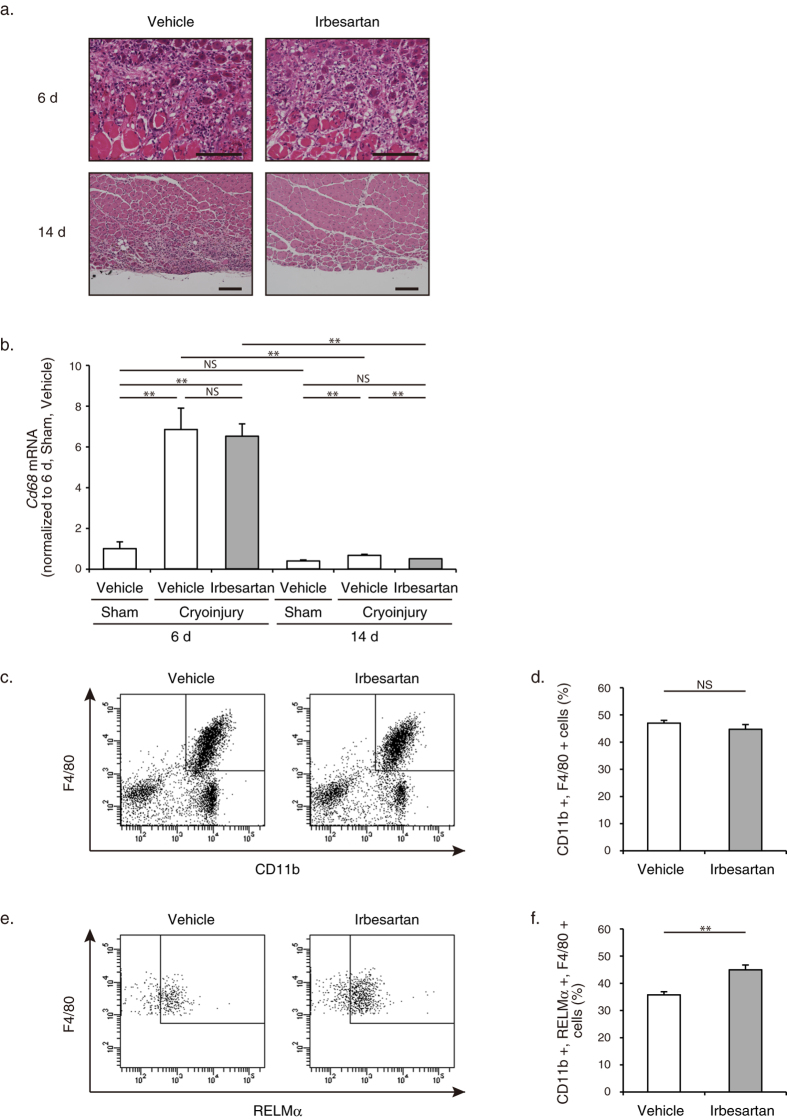
M2 polarization of macrophages in cryoinjured skeletal muscle of irbesartan-treated mice. (**a**) Histological sections with HE staining of TA muscles in mice treated with irbesartan or vehicle at 6 d and 14 d after cryoinjury. Scale bars, 100 μm. (**b**) The mRNA levels of *Cd*68 in TA muscles of mice treated with irbesartan or vehicle at 6 d (*n* = 8, in each group) and 14 d (*n* = 16, in each group) after cryoinjury. Data are shown as fold induction over sham + vehicle at 6 d (mean ± SEM). **P < 0.01. NS, not significant. (**c**) Representative flow cytometric analysis demonstrating CD11b + and F4/80 + macrophages in TA muscles of mice treated with irbesartan or vehicle at 6 d after cryoinjury. (**d**) The percentage of CD11b + and F4/80 + macrophages in flow cytometric analysis of TA muscles of mice treated with irbesartan or vehicle at 6 d after cryoinjury. (*n* = 4, in each group). Data are presented as mean ± SEM. NS, not significant. (**e**) Representative flow cytometric analysis demonstrating F4/80 + and RELMα + M2 macrophages in CD11b + and F4/80 + macrophages sorted from TA muscles of mice treated with irbesartan or vehicle at 6 d after cryoinjury. (**f**) The percentage of CD11b+, F4/80+, and RELMα + M2 macrophages in flow cytometric analysis of TA muscles of mice treated with irbesartan or vehicle at 6 d after cryoinjury (*n* = 4, in each group). Data are presented as mean ± SEM. **P < 0.01.

Macrophages in inflammatory microenvironments have a regulatory role in the muscle response to injury, not only by removing necrotic tissue but also by promoting muscle growth and regeneration^21^. Histological analysis of TA muscles revealed no significant difference at 6 d, but the number of remaining inflammatory cells at 14 d was significantly lower in irbesartan-treated mice than in vehicle-treated mice (Fig. 4a). Consistently, mRNA levels of *Cd*68 encoding the macrophage marker CD68 were comparable in TA muscles at 6 d, but *Cd*68 mRNA levels were significantly lower in irbesartan-treated mice than vehicle-treated mice at 14 d (Fig. 4b), suggesting that inflammatory responses were attenuated in irbesartan-treated mice. Since the M1 to M2 transition of macrophages is important not only for dampening of inflammation, scavenging of debris, and tissue healing, but also for the transition from proliferative to differentiation stages of myogenesis^21^, we examined the polarity of infiltrating macrophages at 6 d. Although the total number of CD11b + and F4/80 + macrophages was unchanged (Fig. 4c,d), flow cytometric analysis revealed that the number of RELMα + M2 macrophages in CD11b + and F4/80 + macrophages was significantly higher in TA muscles at 6 d after cryoinjury of irbesartan-treated mice than in vehicle-treated mice (Fig. 4e,f). These results suggest that AT_1_ receptor blockade induces M2 polarization of macrophages in injured skeletal muscle, and thereby attenuates inflammatory responses and promotes tissue repair.

### Irbesartan decreased C1q mRNA expression in macrophages both *in vivo* and *in vitro*

**Figure 5:**
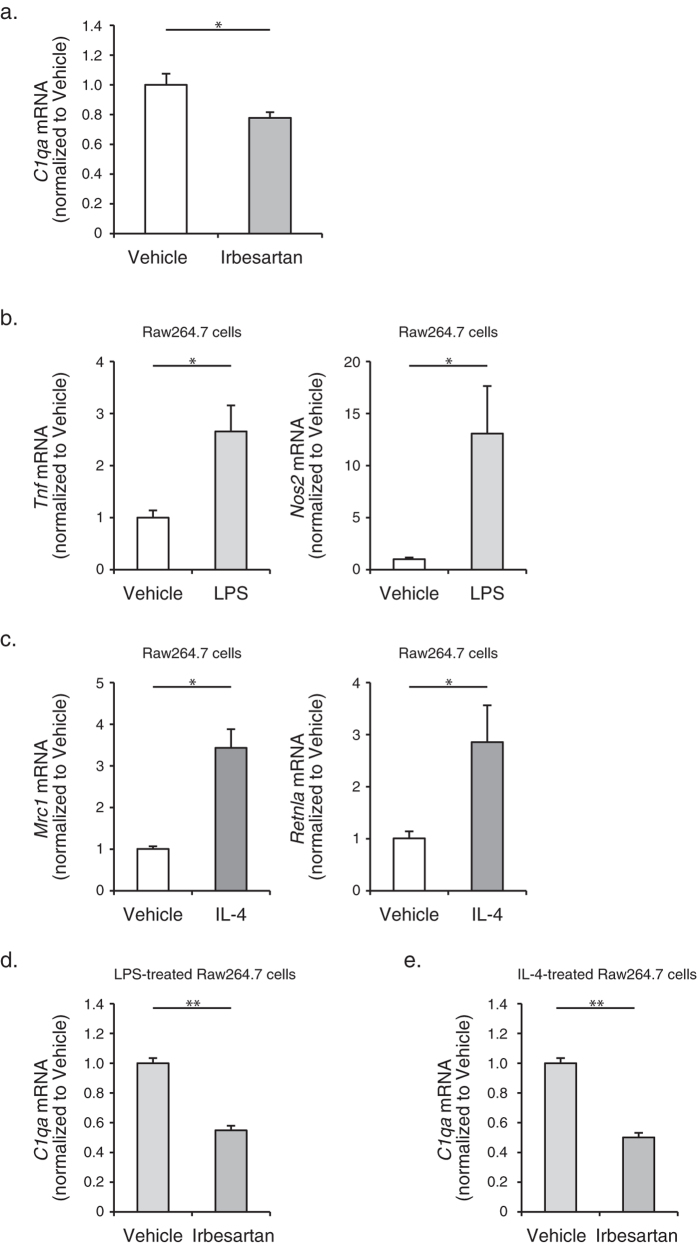
Decreased expression of C1q mRNA expression in macrophages by treatment with irbesartan both *in vivo* and *in vitro.* (**a**) The mRNA levels of *C1qa* in CD11b + and F4/80 + macrophages sorted from TA muscles of mice treated with irbesartan or vehicle at 6 d after cryoinjury (*n* = 6, in each group). Data are shown as fold induction over vehicle (mean ± SEM). *P < 0.05. (**b**) The mRNA levels of *Tnf* and *Nos2* in Raw264.7 cells after stimulation with LPS or vehicle (*n* = 9, in each group). Data are shown as fold induction over vehicle (mean ± SEM). *P < 0.05. (**c**) The mRNA levels of *Mrc1* and *Retnla* in Raw264.7 cells after stimulation with IL-4 or vehicle (*n* = 9, in each group). Data are shown as fold induction over vehicle (mean ± SEM). *P < 0.05. (**d**) The mRNA levels of *C1qa* in LPS-stimulated Raw264.7 cells after treatment with irbesartan or vehicle (*n* = 9, in each group). Data are shown as fold induction over vehicle (mean ± SEM). **P < 0.01. (**e**) The mRNA levels of *C1qa* in IL-4-stimulated Raw264.7 cells after treatment with irbesartan or vehicle (*n* = 9, in each group). Data are shown as fold induction over vehicle (mean ± SEM). **P < 0.01.

Recently, we reported that complement C1q activates canonical Wnt/β-catenin signaling and promotes aging-associated decline in skeletal muscle regeneration^18^. Since macrophages are one of the major sources of C1q biosynthesis^22^, we next explored whether C1q expression was altered by treatment with irbesartan. The mRNA levels of *C1qa* in CD11b + and F4/80 + macrophages sorted from TA muscles at 6 d after cryoinjury were significantly lower in irbesartan-treated mice than in vehicle-treated mice (Fig. 5a). *In vitro*, stimulation with LPS induced M1 polarization in macrophage Raw264.7 cells, as revealed by an increase in mRNA expressions of *Tnf* and *Nos2* (Fig. 5b). In contrast, stimulation with IL-4 induced M2 polarization in Raw264.7 cells, as revealed by an increase in mRNA expressions of *Mrc1* and *Retnla* (Fig. 5c). We found that irbesartan treatment significantly decreased *C1qa* mRNA expression in Raw264.7 cells, when they were either M1-polarized by LPS or M2-pollarized by IL-4 (Fig. 5d,e). These results suggest the hierarchical relationship between AT_1_ receptor signaling and C1q/Wnt-β-catenin signaling.

### Irbesartan suppressed the activation of C1q-Wnt/β-catenin signaling pathway after cryoinjury

**Figure 6:**
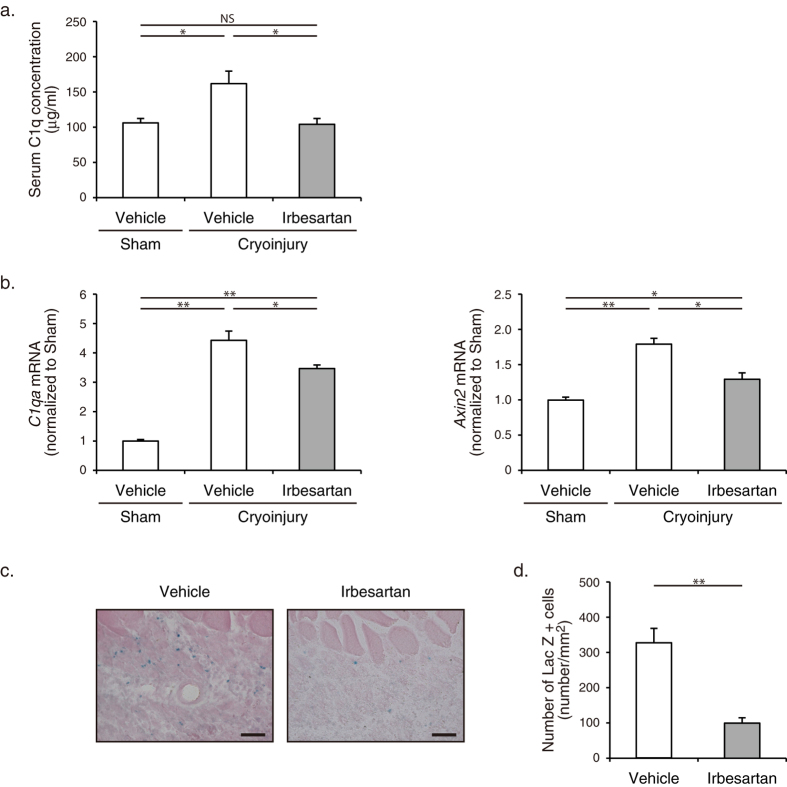
Suppression of cryoinjury-induced activation of C1q-Wnt/β-catenin signaling pathway in irbesartan-treated mice. (**a**) Serum C1q concentrations in mice treated with irbesartan or vehicle at 4 d after cryoinjury or sham operation (*n* = 12, in each group). Data are presented as mean ± SEM. *P < 0.05. NS, not significant. (**b**) The mRNA levels of *C1qa* and *Axin2* in TA muscles of mice treated with irbesartan or vehicle at 4 d after cryoinjury or sham operation (*n* = 9, in each group). Data are shown as fold induction over sham + vehicle (mean ± SEM). *P < 0.05, **P < 0.01. (**c**) Axin2 expression revealed by X-gal staining of TA muscles in *Axin2*^lacZ/+^ mice treated with irbesartan or vehicle at 4 d after cryoinjury. Scale bars, 50 μm. (**d**) The number of LacZ + cells in histological sections of TA muscles in *Axin2*^lacZ/+^ mice treated with irbesartan or vehicle at 4 d after cryoinjury (*n* = 16, in each group). Data are presented as mean ± SEM. **P < 0.01.

Serum C1q concentration was elevated at 4 d after cryoinjury, but irbesartan treatment attenuated the increase in the serum C1q concentration in cryoinjured mice (Fig. 6a). Irbesartan treatment also inhibited the increase in mRNA expressions of *C1qa* and *Axin2*, which is a downstream molecule of the Wnt/β-catenin signaling pathway, in cryoinjured TA muscles at 4 d after cryoinjury, as revealed by real-time RT-PCR analysis (Fig. 6b). X-gal staining also revealed that the number of LacZ + cells in histological sections of TA muscles in *Axin2*^lacZ/+^ mice at 4 d was significantly decreased by irbesartan treatment (Fig. 6c,d). These results suggest that AT_1_ receptor blockade suppresses activation of C1q-Wnt/β-catenin signaling pathway in injured skeletal muscle.

### Topical administration of C1q reversed the beneficial effects of irbesartan on skeletal muscle repair after cryoinjury

**Figure 7:**
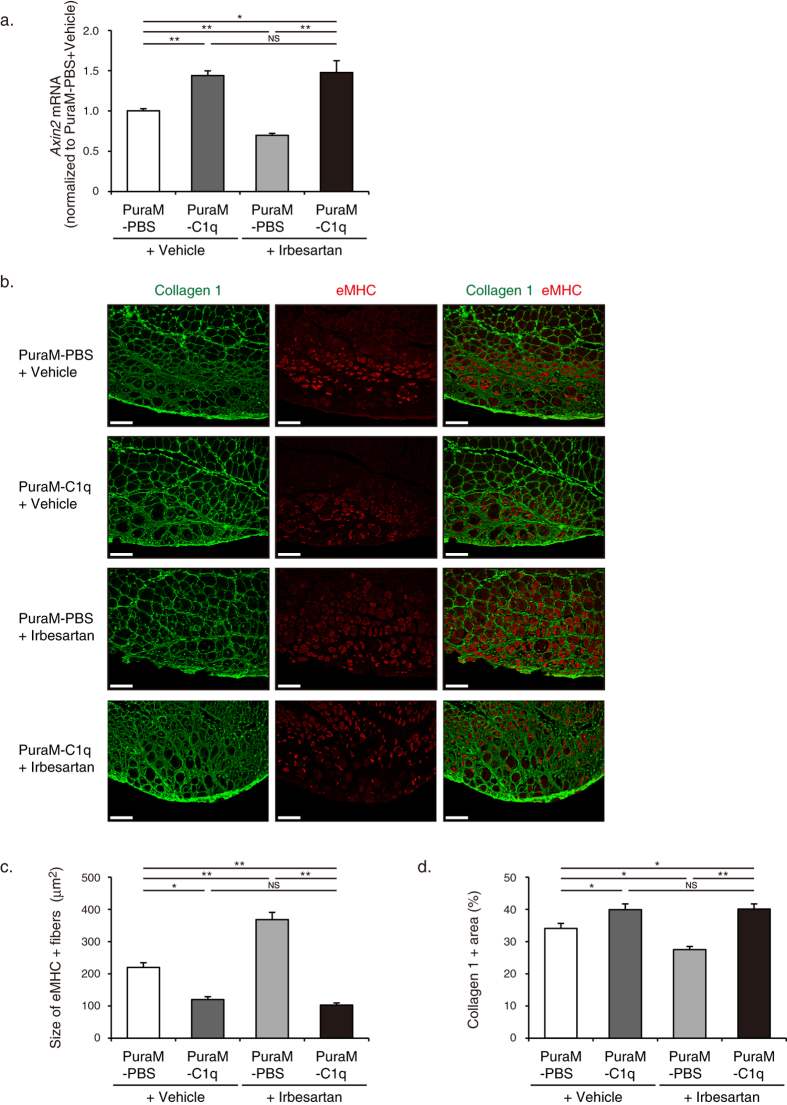
Effects of topical administration of C1q on irbesartan-induced enhancement of skeletal muscle repair after cryoinjury. (**a**) The mRNA levels of *Axin2* in TA muscles of irbesartan- or vehicle-treated mice with topical administration of C1q or PBS in PuraMatrix hydrogel at 2 d after cryoinjury (vehicle + PuraMatrix-PBS, *n* = 12; vehicle + PuraMatrix-C1q, *n* = 7; irbesartan + PuraMatrix-PBS, *n* = 9; irbesartan + PuraMatrix-C1q, *n* = 7). Data are shown as fold induction over vehicle + PuraMatrix-PBS (mean ± SEM). *P < 0.05, **P < 0.01. NS, not significant. PuraM, PuraMatrix. (**b**) Immunostaining of TA muscles of irbesartan- or vehicle-treated mice with topical administration of C1q or PBS in PuraMatrix hydrogel at 10 d after cryoinjury. Embryonic myosin heavy chain (eMHC) and Collagen 1 are represented in red and green, respectively. Scale bar, 100 μm. (**c**) The sizes of eMHC + fibers in TA muscles of irbesartan- or vehicle-treated mice with topical administration of C1q or PBS in PuraMatrix hydrogel at 10 d after cryoinjury (vehicle + PuraMatrix-PBS, *n* = 284; vehicle + PuraMatrix-C1q, *n* = 214; irbesartan + PuraMatrix-PBS, *n* = 338; irbesartan + PuraMatrix-C1q, *n* = 286). Data are presented as mean ± SEM. *P < 0.05, **P < 0.01. NS, not significant. (**d**) The percent area stained for Collagen 1 in TA muscles of irbesartan- or vehicle-treated mice with topical administration of C1q or PBS in PuraMatrix hydrogel at 10 d after cryoinjury (*n* = 7, in each group). *P < 0.05, **P < 0.01. NS, not significant.

Finally, we examined the effects of administration of C1q on irbesartan-induced enhancement of repair and regeneration in TA muscles after cryoinjury. Irbesartan treatment significantly decreased *Axin*2 mRNA expression in TA muscles at 2 d after cryoinjury, but topical administration of C1q in PuraMatrix hydrogel reversed the suppressive effect of irbesartan on *Axin2* mRNA expression (Fig. 7a). Similarly, immunostaining revealed that the increase in the size of eMHC-positive myofibers and the decrease in Collagen 1 + fibrotic area in TA muscles of irbesartan-treated mice at 10 d after cryoinjury were reversed by topical administration of C1q (Fig. 7b–d). These results suggest that AT_1_ receptor blockade improves muscle repair and regeneration through down-regulation of the aging-promoting C1q.

## Discussion

In the present study, we demonstrated that genetic blockade of AT_1_ receptor in mice led to a prolongation of chronological life span (Supplementary Fig. 1), as reported by Benigni A. *et al.*^17^. They described morphological differences in several organs between aged *Agtr1a*^−/−^ mice and wild-type controls, such as lower cardiomyocyte size and collagen deposition in the heart, fewer atherosclerotic lesions in the aorta, and fewer lymphoid aggregates in the pancreas of *Agtr1a*^−/−^ mice^17^. Our analysis revealed that aging-related decline in skeletal muscle function was remarkably milder in *Agtr1a*^−/−^ mice (Supplementary Fig. 3). One of the most important aging-related features in our body is the locomotive decline, which has a great impact on individual health span and quality of life. Locomotive decline is primarily caused by skeletal muscle aging, which is characterized by the loss of muscle mass and strength^23^. The life-long maintenance of skeletal muscle is ensured by the continuous and balanced renewal of myofibers^19^. Therefore, we investigated the role of AT_1_ receptor signaling in skeletal muscle regeneration by applying cryoinjury on TA muscles. Our analysis revealed that irbesartan significantly enhanced regenerative capacity of skeletal muscle with better muscular differentiation and growth, and that it induced M2 polarization of macrophages and down-regulated aging-promoting C1q expression. According to an observational study, elderly women with hypertension continuously using Ang converting enzyme (ACE) inhibitors showed a lower decline in muscle strength than intermittent or never users of ACE inhibitors^24^. Cross-sectional analysis also demonstrated that the use of ACE inhibitors was associated with larger muscle mass of lower extremities in the elderly^25^. Our study may provide mechanistic insights into the beneficial effects of pharmacological inhibition of renin-angiotensin system in the prevention of age-related sarcopenia.

To explore the impact of AT_1_ receptor blockade on skeletal muscle regeneration, we inhibited AT_1_ receptor activation in cryoinjured mice by treatment with irbesartan, not by genetic disruption of *Agtr1a* gene, to eliminate possible metabolic effects of hyperphagia and obesity in *Agtr1a*^−/−^ mice^20^. Irbesartan is one of the ARBs that are commercially available as highly effective and well-tolerated drugs for the management of hypertension^26^,^27^. Although peripheral administration of irbesartan was reported to have access to central nervous system^28^, we observed that irbesartan, orally administered at a subpressor dose, showed no significant effect on daily food intake and body weight (Supplementary Fig. 4). There has been conflicting evidence for AT_1_ receptor blockade on skeletal muscle regeneration^29^. Bedair HS. *et al.* and Burks TN. *et al.* reported that treatment with the ARB losartan in drinking water led to histological improvement in muscle regeneration after laceration-induced injury in mice^30^,^31^, but in contrast, Johnston APW. *et al.* reported that treatment with the angiotensin-converting enzyme inhibitor captopril or genetic disruption of *Agtr1a* gene led to significant impairment in muscle growth after cardiotoxin-induced injury in mice^32^. Meanwhile, Murphy KT. *et al.* reported that *Agtr1a*^−/−^ mice exhibited impaired muscle regeneration after notexin-induced injury, while they had enhanced muscle strength, mobility, and locomotor activity at baseline^33^. Although the reasons for these discrepant results are currently unclear, irbesartan, in our hands, promoted repair and regeneration of skeletal muscle after cryoinjury (Figs 1 and 2). Besides selectively binding to the AT_1_ receptor and inhibiting Ang II-induced receptor activation, irbesartan has been reported to exert pleiotrophic actions such as activation of peroxisome proliferator-activated receptor-γ^34^,^35^ and inhibition of nuclear factor-κB activity^36^. Further studies will be required to elucidate whether the actions independent of the AT_1_ receptor inhibition make significant contributions to the beneficial effects of irbesartan on skeletal muscle repair and regeneration.

Regenerative capacity of skeletal muscle could be caused by perturbations to the processes that regulate activation, proliferation, and differentiation of satellite cells^21^. In response to injury, neutrophils and M1 macrophages infiltrate the damaged muscles and release proinflammatory cytokines, leading to further damage of myofibers and activation and proliferation of satellite cells. Subsequently, M1 macrophages are replaced by a population of M2 macrophages that attenuate the inflammatory response and promote differentiation and growth of satellite cells^21^. Therefore, myogenic regeneration is tightly linked to inflammatory microenvironments. We observed that treatment with irbesartan promoted repair and growth of skeletal muscle after cryoinjury (Fig. 1). Irbesartan also induced M2 polarization of macrophages in injured muscles (Fig. 4), which was consistent with the previous findings that treatment with ARB skewed intrarenal macrophages from M1 to M2 phenotype in a mouse model of obesity-related kidney injury^37^ and a rat model of anti-glomerular basement membrane glomerulonephritis^38^. Although little is known about the mechanisms how AT_1_ receptor blockade induces M2 polarization of macrophages, the shift in macrophages from M1 to M2 phenotype might contribute to the beneficial effects of irbesartan on muscle growth and repair after cryoinjury.

Our analysis indicated that irbesartan promoted activation and proliferation of Pax7 + satellite cells following injury (Fig. 3). It has been reported that the proliferative potential declined with aging because of an increase in transforming growth factor-β (TGF-β) signaling and a decrease in Notch signaling pathway^39^, and that AT_1_ receptor blockade suppressed TGF-β signaling, and thereby attenuated TGF-β-mediated impairment of muscle regeneration in myopathic mice with Fibrillin 1 mutation or dystrophin deficiency^40^. Since AT_1_ receptor is expressed in satellite cells, AT_1_ receptor blockade may also inhibit the direct anti-proliferative actions of Ang II on satellite cells^41^. In the present study, we demonstrated that down-regulation of the aging-promoting C1q mediated beneficial effects of AT_1_ receptor blockade on muscle repair and regeneration (Figs 5, 6, 7). We recently identified C1q as a diffusible factor in the serum that activates canonical Wnt/β-catenin signaling by binding to Wnt receptor Frizzled and by inducing cleavage of Wnt coreceptor LRP6^18^. Serum and tissue concentrations of C1q showed a significant increase with aging, and C1q-mediated activation of Wnt/β-catenin signaling led to aging-related decline in regeneration capacity of skeletal muscles^18^. C1q treatment attenuated satellite cell proliferation and stimulated fibroblast proliferation both *in vitro* and *in vivo*^18^. We also reported that M2 macrophages were recruited to the aorta and secreted C1q, which promoted arterial remodeling through activation of Wnt/β-catenin signaling, in Ang II-infused mice^42^. We propose that AT_1_ receptor blockade reduces systemic and local levels of C1q through inhibiting C1q production by infiltrated macrophages, and thereby enhances activation and proliferation of satellite cells. Insomuch as AT_1_ receptor is expressed in both myocytes and macrophages, further studies will be required to elucidate the cell type-specific role of AT_1_ receptor in inducing C1q expression in macrophages in injured muscles.

In conclusion, AT_1_ receptor blockade prolongs life span, and improves muscle repair and regeneration through modulating the inflammatory microenvironment, including M2 polarization of macrophages and down-regulation of the aging-promoting C1q-Wnt/β-catenin signaling pathway. Elucidation of detailed relationship between AT_1_ receptor signaling and C1q/Wnt-β-catenin signaling will be of interest, and will contribute to understanding the mechanisms of functional decline of multiple organs with aging and developing preventive and therapeutic measures against aging-related diseases.

## Methods

### Mice, and cryoinjury

All of the experiments were approved by the Institutional Animal Care and Use Committees of Osaka University and Chiba University, and carried out in accordance with the guidelines of Osaka University and Chiba University. C57BL/6J mice were purchased from CLEA Japan, Inc., and generation of *Agtr1a*^−/−^ mice and *Axin2*^lacZ/+^ mice has been described previously^43^,^44^. The mice were fed a standard chow (CRF-1; Charles River Laboratories Japan, Inc.) or a chow containing irbesartan (Shionogi & Co., Ltd.). Treatment with irbesartan (20 mg/kg/day) was started one day prior to cryoinjury operation, and continued until cessation of the experiment. To produce cryoinjury, we anesthetized 7 to 10-week-old male mice by intraperitoneal injection of medetomidine hydrochloride (0.3 mg/kg), midazolam (4 mg/kg), and butorphanol (5 mg/kg)^45^, and anesthesia was monitored by pinching the toe. We cooled a metal probe (2 mm × 10 mm in dimensions) in liquid nitrogen, and applied it directly onto the exposed TA muscle of mice for 10 s, as described previously^46^. Post-operative analgesia (meloxicam, 5 mg/kg/24 h) was administered subcutaneously for 48 h. The surgeon had no information about the mice used in this study.

### Blood pressure measurement

The systolic and diastolic blood pressures and pulse rates were measured in conscious mice noninvasively by a programmable sphygmomanometer (BP-98A; Softron) using the tail-cuff method.

### Analysis of hair growth

Age-matched young (12 to 16-week-old) and old (72-week-old) mice were shaved on their dorsal surface (2.0 cm^2^) using an electric razor for hair grow assay, as described previously^47^. We measured an area with hair growth 28 d after shaving.

### Analysis of skeletal muscle function

A vertical pole test was used to test the motor balance of mice, as described previously^48^. A mouse was placed onto a horizontally positioned wood pole (2 cm in diameter and 50 cm long), and the pole was raised gently and slowly to a 90° position. The length of time for the mouse to fall off the pole was recorded. A hanging wire test was used to test the muscle strength, tone, and equilibrium, as described previously^49^. A mouse was placed onto a horizontally positioned wire (1.5 mm in diameter and 15 cm in length), and the wire was placed 30 cm above the floor. The mouse was required to grasp the middle part of the wire with their forepaws, and the length of time for the mouse to fall off the wire was recorded. A cutoff time of 1 min was used for the vertical pole and hanging wire tests. A treadmill test was used to assess running performance. Mice were subjected to a horizontal treadmill at 13 m/min after acclimatization to a rodent treadmill device (KN-73; Natsume Seisakusho, Co. Ltd.) by 5 min rest on the conveyor belt and subsequent 5 min running. Running was terminated when mice touched the electric shock grid at the back of the treadmill more than 10 times/min, and the total running distance was recorded.

### Cell culture

Raw264.7 cells (American Type Culture Collection) were plated at a density of 1 × 10^5^ cells/3.5 cm dish in Dulbecco’s modified Eagle’s medium supplemented with 10% fetal bovine serum. After 12 h of culture, Raw264.7 cells were stimulated with 0.5 ng/ml of LPS (Sigma-Aldrich) or 0.4 ng/ml of IL-4 (PeproTech, Inc.) for 24 h and then treated with 10^−7^ M of irbesartan or vehicle for 3 h.

### Histological analysis

For histological analysis, TA muscles were excised, fixed in 10% neutralized formalin, and embedded in paraffin. Serial sections at 5 μm were deparaffinized and stained with hematoxylin and eosin for morphological analysis and Masson’s trichrome for evaluation of fibrosis. Images were acquired with a microscope (FSX100; Olympus), and for measurement of the cross-sectional area of centronuclear myofibers, images of random fields of injured area were analyzed using WinROOF software (Mitani Corporation). We evaluated a ratio of fibrotic area to injured area for quantification of the fibrotic area (%) in images acquired with a microscope (BZ-X700; Keyence Corporation) using BZ-X analysis application (Keyence Corporation).

### Immunohistochemical analysis

*In vivo* cryotechnique was used to freeze TA muscles promptly for immunohistochemical analysis^50^. TA muscles of anesthetized mice were exposed, and frozen by directly pouring liquid isopentane-propane cryogen (−193 °C) precooled in liquid nitrogen. The frozen muscles were removed with an electric dental drill in liquid nitrogen, and processed for freeze-substitution fixation in acetone containing 0.2% glutaraldehyde at −80 °C for 24 h and then −30 °C and 4 °C for 2 h each. After being left at room temperature for 1 h, they were washed in acetone, immersed in 30% sucrose overnight, and frozen with isopentane precooled in dry ice. For immunofluorescence, cryostat sections (7 μm) were fixed in acetone, were incubated with the following primary antibodies overnight at 4 °C: mouse monoclonal anti-eMHC antibody (clone F1.652, Developmental Studies Hybridoma Bank), rabbit polyclonal anti-Collagen 1 antibody (Abcam), mouse monoclonal anti-Pax7 antibody (clone #PAX7, R&D Systems, Inc.), and rat monoclonal anti-Laminin 2 alpha antibody (clone 4H8-2, Abcam). Secondary antibodies (Alexa Fluoro 488-conjugated anti-rabbit IgG antibody, Alexa Fluoro 488-conjugated anti-rat IgG antibody, or Alexa Fluoro 594-conjugated anti-mouse IgG antibody (Life Technologies, Inc.)) and TO-PRO-3 (Life Technologies, Inc.) were applied to visualize expression of specific proteins and nuclei, respectively. Sections were mounted with ProLong Gold Antifade Reagent (Life Technologies, Inc.). Images were acquired with either a fluorescence microscope (BZ-X700; Keyence Corporation) or an LSM 700 confocal microscope (Carl Zeiss), and analyzed using WinROOF software (Mitani Corporation).

### X-gal staining

X-gal stainings were performed, as described previously^51^. TA muscles were excised from 10-week-old *Axin2*^lacZ/+^ mice 4 d after cryoinjury. Cryostat sections (10 μm) were stained with X-gal, and counterstained with nuclear fast red.

### Real time RT-PCR analysis

Total RNA was extracted by using the TRIzol Reagent (Life Technologies, Inc.), and treated with DNase to remove contaminating genomic DNA using TURBO DNA-free Kit (Life Technologies, Inc.). Single-stranded cDNA was transcribed by using The SuperScript VILO cDNA Synthesis Kit (Life Technologies, Inc.) according to the manufacturer’s protocol. We conducted quantitative real-time PCR analysis using Light Cycler TaqMan Master Kit (Roche Applied Science) with the target-specific primers and the matching probes designed by the Universal ProbeLibrary System (Roche Applied Science), according to the manufacturer’s instructions. Amplification conditions were initial denaturation for 10 min at 95 °C followed by 45 cycles of 10 s at 95 °C and 25 s at 60 °C. Individual PCR products were analyzed by melting-point analysis. The expression level of a gene was normalized relative to that of mouse *Gapdh* by using a comparative Ct method. The primer sequences and Universal Probe numbers were designed with the ProbeFinder software as following: *Tgfb1*, 5′-tggagcaacatgtggaactc-3′ and 5′-cagcagccggttaccaag-3′, No. 72; *Postn*, 5′-cgggaagaacgaatcattaca-3′ and 5′-ttgcaggtgtgtctttttgc-3′, No. 10; *Col1a1*, 5′-agacatgttcagctttgtggac-3′ and 5′-gcagctgacttcagggatg-3′, No.15; *Col3a1*, 5′-tcccctggaatctgtgaatc-3′ and 5′-tgagtcgaattggggagaat-3′, No. 49; *Cd68*, 5′-cgccatgaatgtccactg-3′ and 5′-gacctacatcagagcccgagt-3′, No. 96; *C1qa,* 5′-gggtctcaaaggagagagg-3′ and 5′-tcctttaaaacctcggaacca -3′, No. 17; *Tnf*, 5′-tcttctcattcctgcttgtgg-3′ and 5′-ggtctgggccatagaactga-3′, No. 49; *Nos2*, 5′-gggctgtcacggagatca-3′ and 5′-ccatgatggtcacattctgc-3′, No. 76; *Mrc1*, 5′-ccacagcattgaggagtttg-3′ and 5′-acagctcatttggctca-3′, No. 7; *Retnla,* 5′-ccctccactgtaacgaagactc-3′ and 5′-cacacccagtagcagtcatcc-3′, No. 51; *Axin2,* 5′- gagagtgagcggcagagc-3′ and 5′-cggctgactcgttctcct-3′, No. 96; *Gapdh*, 5′-tgtccgtcgtggatctgac- 3′ and 5′-cctgcttcaccaccttcttg-3′, No. 80.

### Flow cytometric analysis

TA muscles were minced and digested in high glucose Dulbecco’s modified Eagle’s medium containing 0.2% collagenase type 1 (Wako Pure Chemical Industries, Ltd.). After digested tissues were further dissociated with 18 G needle and remaining debris was sedimented, the supernatant was collected after filtering through 100- and 40-μm cell strainer (Corning, Inc.), and cells were suspended in PBS containing 3% fetal bovine serum. After Fc receptor blocking using rat anti-mouse CD16/CD32 monoclonal antibody (clone 2.4G2, BD Biosciences), cells were incubated with PE-conjugated anti-F4/80 antibody (Biolegend, Inc.) and PerCP-Cy5.5-conjugated anti-CD11b antibody (BD Biosciences) for 30 min on ice, and washed with PBS containing 3% fetal bovine serum. Dead cells were excluded using LIVE/DEAD Fixable Dead Cell Stain Kit (Life Technologies, Inc.). The percentages of CD11b + and F4/80 + cells were analyzed by the BD FACSAria II (BD Biosciences) using BD FACSDiva software (BD Biosciences). CD11b + and F4/80 + cells were sorted and collected using the BD FACSAria II (BD Biosciences) for real time RT-PCR analysis and further staining with RELMα. CD11b + and F4/80 + cells were fixed and permeabilized in BD Cytofix/Cytoperm buffer (BD Biosciences) for 20 min on ice, and washed in BD Perm/Wash solution (BD Biosciences). Cells were incubated with biotinylated rabbit polyclonal anti-RELMα antibody (Abcam) for 30 min on ice, followed by APC-conjugated streptavidin (BD Biosciences) for 30 min on ice, and washed with PBS containing 3% fetal bovine serum. The percentages of CD11b + and RELMα + cells were analyzed by the BD FACSAria II (BD Biosciences) using BD FACSDiva software (BD Biosciences).

### ELISA for C1q

Serum C1q concentration was determined by using C1q, Mouse, ELISA kit (Hycult Biotech), according to the manufacturer’s protocol.

### Statistical analysis

All of the data are presented as mean ± SEM. Two-group comparison was analyzed by unpaired 2-tailed Student’s *t* test and Welch’s *t* test, and multiple-group comparison was performed by1-way ANOVA followed by the Tukey-Kramer HSD test and Steel-Dwass test for comparison of means. We estimated survival curves by the Kaplan-Meier method, and compared the groups using the log-rank test. Values of *P* < 0.05 were considered statistically significant.

## Additional Information

**How to cite this article**: Yabumoto, C. *et al.* Angiotensin II receptor blockade promotes repair of skeletal muscle through down-regulation of aging-promoting C1q expression. *Sci. Rep.* **5**, 14453; doi: 10.1038/srep14453 (2015).

## Supplementary Material

Supplementary Information
