# Supplementary material for: Angiotensin II receptor blockade promotes repair of skeletal muscle through down-regulation of aging-promoting C1q expression
Source: Sci Rep. 2015 Sep 25;5:14453. doi: 10.1038/srep14453 (PMC4585890; doi:10.1038/srep14453)
Supplement: Supplementary Information [file srep14453-s1.pdf]

Supplementary information

Angiotensin II receptor blockade promotes repair of skeletal muscle through down-regulation of aging-promoting C1q expression

Chizuru Yabumoto, Hiroshi Akazawa, Rie Yamamoto, Masamichi Yano, Yoko Kudo-Sakamoto, Tomokazu Sumida, Takehiro Kamo, Hiroki Yagi, Yu Shimizu, Akiko Saga-Kamo, Atsuhiko T. Naito, Toru Oka, Jong-Kook Lee, Jun-ichi Suzuki, Yasushi Sakata, Etsuko Uejima, Issei Komuro

Supplementary Table 1. Heart rates and blood pressures of irbesartan- or vehicle-treated mice

	0 d		14 d	
	Vehicle	Irbesartan	Vehicle	Irbesartan
Number	6	6	6	6
Heart rate (bpm)	640.2 ± 33.5	674.8 ± 15.2	695.5 ± 16.9	688.3 ± 11.7
SBP (mmHg)	102.2 ± 0.9	101.2 ± 0.8	102.7 ± 0.7	102.8 ± 2.0
DBP (mmHg)	59.7 ± 3.9	56.8 ± 5.1	65.0 ± 1.5	61.5 ± 5.9
MBP (mmHg)	71.8 ± 1.8	71.4 ± 2.9	76.6 ± 1.7	73.8 ± 3.4

Values are mean ± SEM. SBP, systolic blood pressure; DBP, diastolic blood pressure; MBP, mean blood pressure.

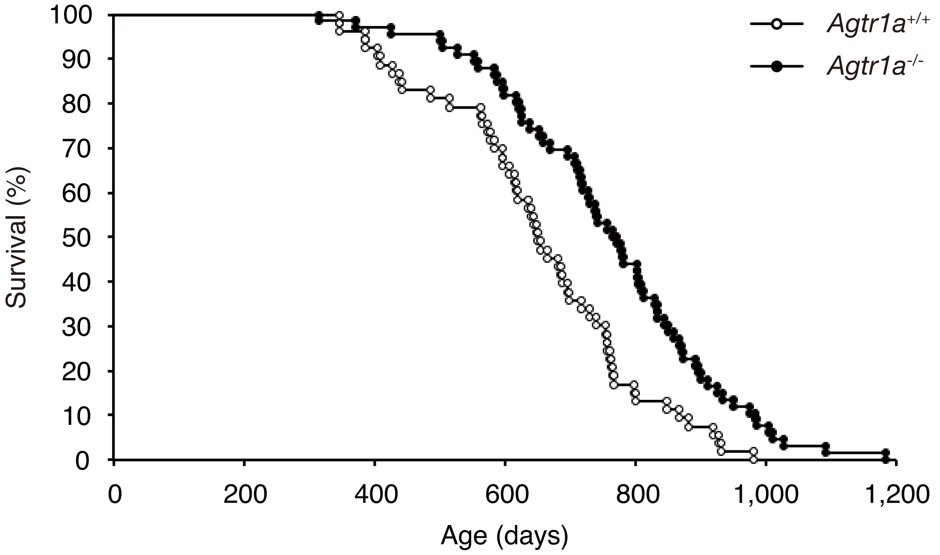

Supplementary Figure 1. Genetic disruption of *Agtr1a* extended life span in mice.

Kaplan-Meier survival curves of *Agtr1a*$^{-/-}$ (n = 66) and *Agtr1a*$^{+/+}$ (n = 55) mice. The average life span of *Agtr1a*$^{-/-}$ and *Agtr1a*$^{+/+}$ mice was 760.0 $\pm$ 20.9 and 651.8 $\pm$ 21.7 days, respectively ($P < 0.05$).

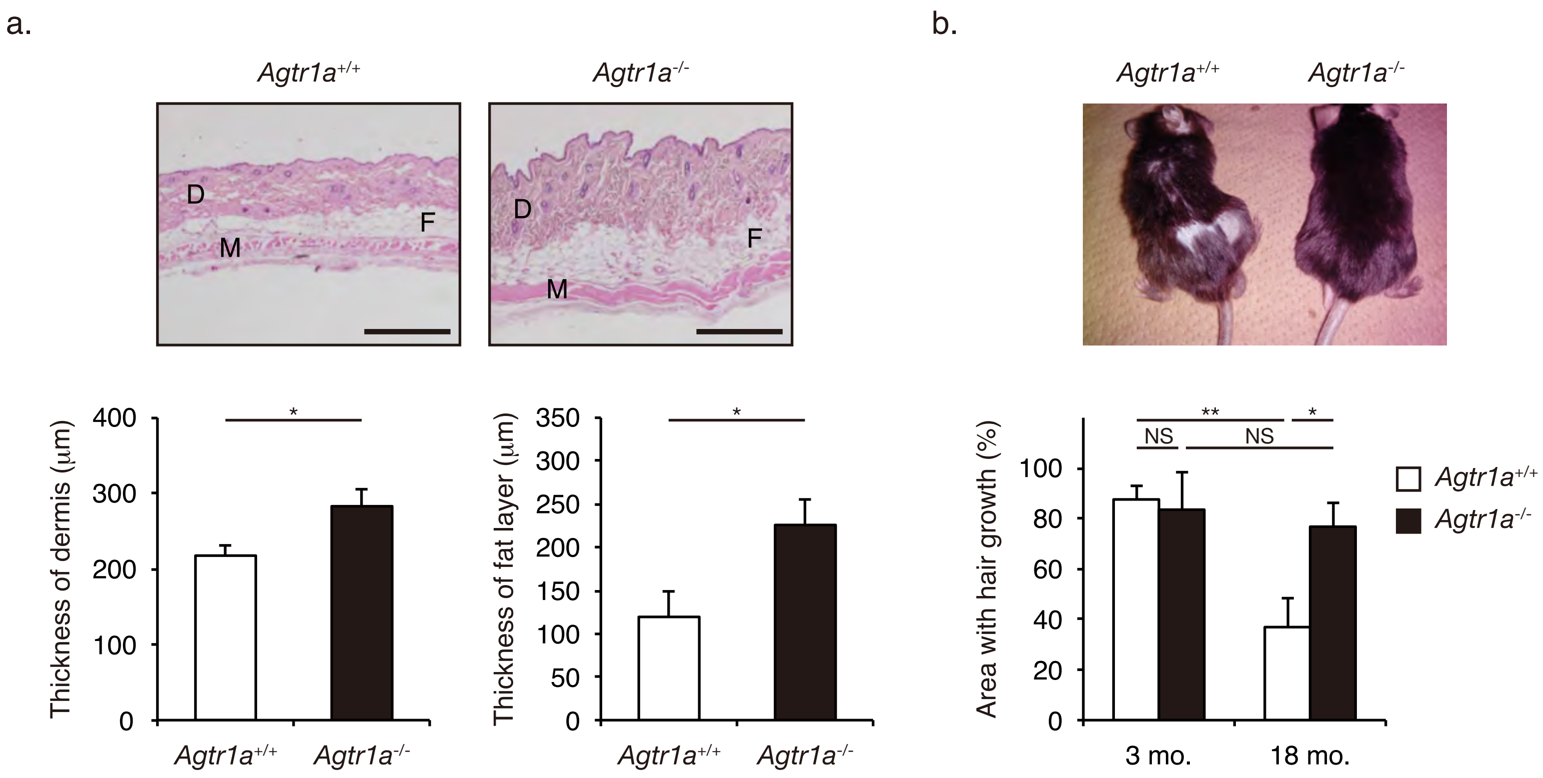

Supplementary Figure 2. Aging-related changes in the skin of *Agtr1a*$^{-/-}$ and *Agtr1a*$^{+/+}$ mice.

(a) Representative images of hematoxylin-eosin staining (upper panels) and the thickness of dermis and fat layer (lower panels, $n = 8$ in each group) in 21-month-old *Agtr1a*$^{-/-}$ and *Agtr1a*$^{+/+}$ mice. F, fat; D, dermis; M, muscularis. $*P < 0.05$. Scale bars, 500 μm.

(b) Representative photos of 18-month-old *Agtr1a*$^{-/-}$ and *Agtr1a*$^{+/+}$ mice at 28 d after shaving of hair from a 2 cm^2 dorsal area (upper panels) and the fraction of hair growth in 3- and 18-month-old *Agtr1a*$^{-/-}$ and *Agtr1a*$^{+/+}$ mice (lower panel) (3-month-old *Agtr1a*$^{+/+}$ mice, $n = 5$; 3-month-old *Agtr1a*$^{-/-}$ mice, $n = 6$; 18-month-old *Agtr1a*$^{+/+}$ mice, $n = 9$; 18-month-old *Agtr1a*$^{-/-}$ mice, $n = 11$). $*P < 0.05$, $**P < 0.01$, NS, not significant.

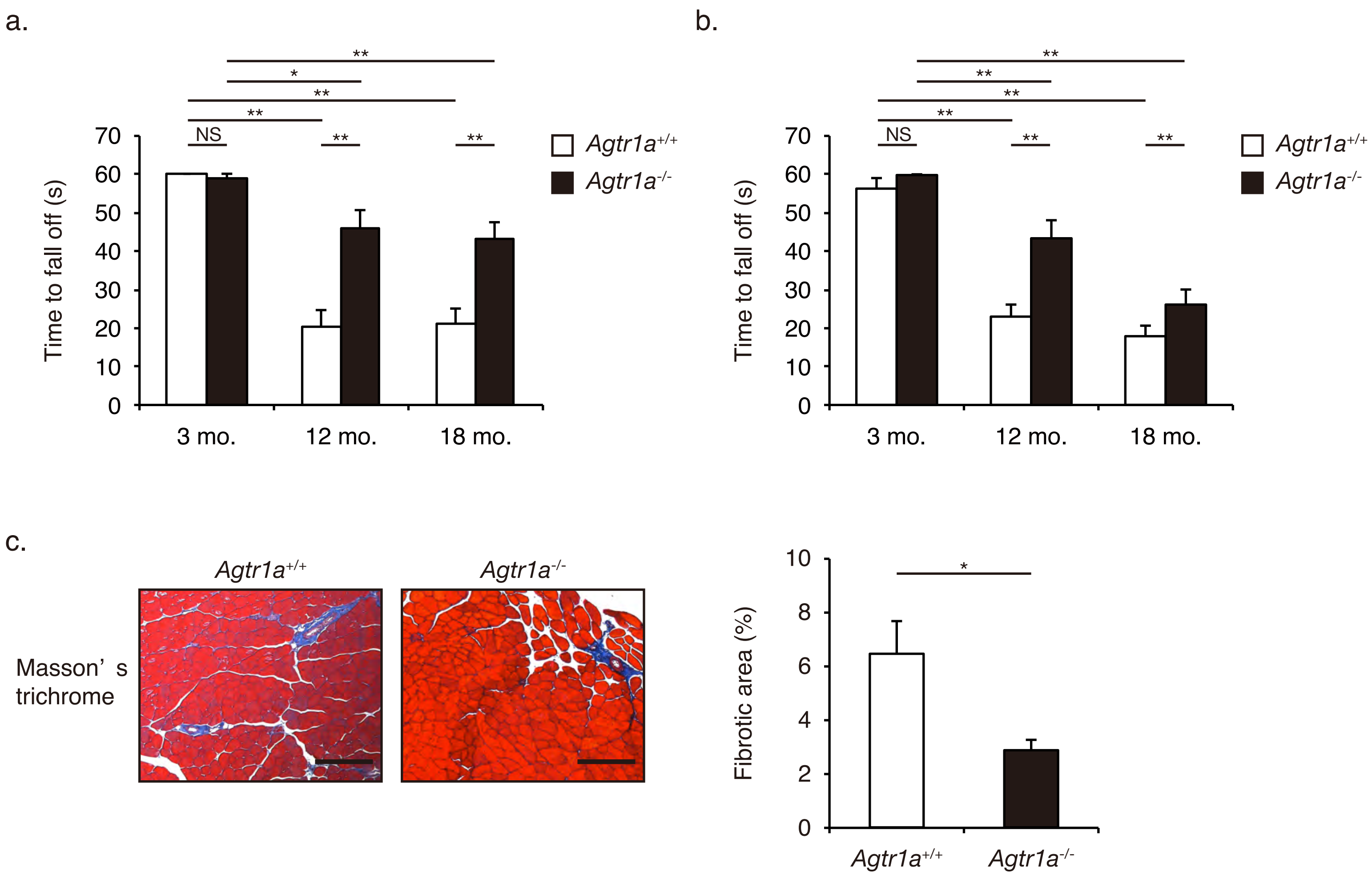

Supplementary Figure 3. Aging-related changes in the skeletal muscle structure and function of *Agtr1a*$^{-/-}$ and *Agtr1a*$^{+/+}$ mice.

(a) The comparison of motor balance using a vertical pole test in 3-, 12-, and 18-month-old *Agtr1a*$^{-/-}$ and *Agtr1a*$^{+/+}$ mice (3-month-old *Agtr1a*$^{+/+}$ mice, $n = 7$; 3-month-old *Agtr1a*$^{-/-}$ mice, $n = 7$; 12-month-old *Agtr1a*$^{+/+}$ mice, $n = 14$; 12-month-old *Agtr1a*$^{-/-}$ mice, $n = 12$; 18-month-old *Agtr1a*$^{+/+}$ mice, $n = 17$; 18-month-old *Agtr1a*$^{-/-}$ mice, $n = 17$). $^{*}P < 0.05$, $^{**}P < 0.01$, NS, not significant.

(b) The comparison of muscular strength, tone, and equilibrium using a hanging wire test in 3-, 12-, and 18-month-old *Agtr1a*$^{-/-}$ and *Agtr1a*$^{+/+}$ mice (3-month-old *Agtr1a*$^{+/+}$ mice, $n = 7$; 3-month-old *Agtr1a*$^{-/-}$ mice, $n = 7$; 12-month-old *Agtr1a*$^{+/+}$ mice, $n = 14$; 12-month-old *Agtr1a*$^{-/-}$ mice, $n = 12$; 18-month-old *Agtr1a*$^{+/+}$ mice, $n = 17$; 18-month-old *Agtr1a*$^{-/-}$ mice, $n = 17$). $^{**}P < 0.01$, NS, not significant.

(c) Histological sections with Masson' s trichrome staining of TA muscles in approximately 24-month-old *Agtr1a*$^{-/-}$ and *Agtr1a*$^{+/+}$ mice. Right panel indicates the percent area of fibrosis in Masson' s trichrome staining of TA muscles (*Agtr1a*$^{+/+}$ mice, $n = 6$; *Agtr1a*$^{-/-}$ mice, $n = 6$). Scale bars, 200 μm, $^{*}P < 0.05$.

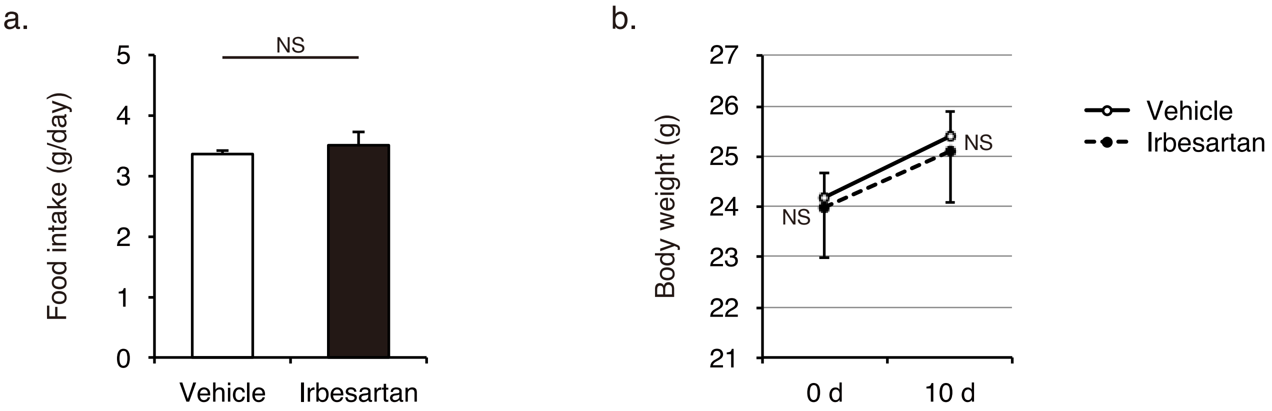

Supplementary Figure 4. Peripherally administered irbesartan had no effect on food intake and body weight.

(a) Daily food intake of irbesartan- or vehicle-treated mice (*n* = 5, in each group). NS, not significant.

(b) Body weight of mice at 0 d and 10 d after treatment with irbesartan or vehicle (*n* = 5, in each group). NS, not significant.
